# The Accuracy of Predictive Analytics in Forecasting Emergency Department Volume Before and After Onset of COVID-19

**DOI:** 10.5811/westjem.61059

**Published:** 2023-12-08

**Authors:** Anthony M. Napoli, Rachel Smith-Shain, Timmy Lin, Janette Baird

**Affiliations:** Brown University, Alpert School of Medicine, Department of Emergency Medicine, Providence, Rhode Island

## Abstract

**Introduction:**

Big data and improved analytic techniques, such as triple exponential smoothing (TES), allow for prediction of emergency department (ED) volume. We sought to determine 1) which method of TES was most accurate in predicting pre-coronavirus 2019 (COVID-19), during COVID-19, and post-COVID-19 ED volume; 2) how the pandemic would affect TES prediction accuracy; and 3) whether TES would regain its pre-COVID-19 accuracy in the early post-pandemic period.

**Methods:**

We studied monthly volumes of four EDs with a combined annual census of approximately 250,000 visits in the two years prior to, during the 25-month COVID-19 pandemic, and the 14 months following. We compared the accuracy of four models of TES forecasting by measuring the mean absolute percentage error (MAPE), mean square errors (MSE) and mean absolute deviation (MAD), comparing actual to predicted monthly volume.

**Results:**

In the 23 months prior to COVID-19, the overall average MAPE across four forecasting methods was 3.88% ± 1.88% (range 2.41–6.42% across the four ED sites), rising to 15.21% ± 6.67% during the 25-month COVID-19 period (range 9.97–25.18% across the four sites), and falling to 6.45% ± 3.92% in the 14 months after (range 3.86–12.34% across the four sites). The 12-month Holt-Winter method had the greatest accuracy prior to COVID-19 (3.18% ± 1.65%) and during the pandemic (11.31% ± 4.81%), while the 24-month Holt-Winter offered the best performance following the pandemic (5.91% ± 3.82%). The pediatric ED had an average MAPE more than twice that of the average MAPE of the three adult EDs (6.42% ± 1.54% prior to COVID-19, 25.18% ± 9.42% during the pandemic, and 12.34% ± 0.55% after COVID-19). After the onset of the pandemic, there was no immediate improvement in forecasting model accuracy until two years later; however, these still had not returned to baseline accuracy levels.

**Conclusion:**

We were able to identify a TES model that was the most accurate. Most of the models saw an approximate four-fold increase in MAPE after onset of the pandemic. In the months following the most severe waves of COVID-19, we saw improvements in the accuracy of forecasting models, but they were not back to pre-COVID-19 accuracies.

Population Health Research Capsule What do we already know about this issue?

*Predictive analytics are more accurate than subjective expert opinion for forecasting future events. However, the accuracy may be compromised by large-scale, abrupt disruptive events.*

What was the research question?

*Was the accuracy of forecasting methods for ED volume disrupted by the COVID-19 pandemic?*

What was the major finding of the study?

*Predictive models accuracy changed from mean absolute percentage errors of 3.18% ± 1.65% pre-pandemic to 11.31% ± 4.81% after onset of COVID-19.*

How does this improve population health?

*While abrupt disruptive events such as a pandemic may affect the accuracy of models predicting ED volume, accuracy will improve over time.*

## INTRODUCTION

Forecasting emergency department (ED) volume is critical to determining staffing needs and operational planning. Forecasting methodologies for predicting future volume have historically relied on subjective predictions paired with historical volume. However, in recent years more sophisticated methods of forecasting have been employed by pairing large-scale data availability with newer predictive analytics techniques.^1^ ^,^ ^2^ Variations in ED volume due to seasonal and day of the week fluctuation have a general pattern that can be predicted based on advanced analytical techniques.^3^ ^–^ ^5^ The benefits of advanced predictive capacity include calibrating staffing to volume needs, revising labor resources with operational demands, infrastructure planning, and informing financial planning.

Various methodologies exist for attempting to predict ED volume; however, linear regression models have shown the most promise.^4^ ^,^ ^6^ Such methods have been shown to predict ED volumes with low mean absolute percentage errors (MAPE). One study of four hospitals in Paris, France, found a MAPE of 5%^6^ while a Dutch study found a MAPE of 8.7%.^5^ Another study that incorporated less conventional time-series techniques found a MAPE of 8–10%.^7^ A fourth study in two Chinese EDs used a hybrid method to obtain MAPEs in the range of 5%.^8^ Lastly, one study using internet search data showed improved model accuracy when including atmospheric data and weather patterns.^9^

Triple exponential smoothing (TES) has become one of the most recognized, reliable methods of predictive analytics for anticipating unknown volumes. While methods like TES are likely more accurate than subjective volume estimates, they are predicated on the assumption of similarity between recent experience and future expectations. Highly variable periods brought on by times of extreme uncertainty, such as the COVID-19 pandemic, raise questions about how predictive analytics based on historical results would perform. We sought to determine 1) which method of TES was most accurate in predicting pre-COVID-19, during COVID-19 and post COVID-19 ED volumes; 2) what would the effect of the pandemic be on exponential smooth accuracy; and 3) whether such models could regain their pre-COVID-19 accuracy after the disruptive influence of COVID-19.

## METHODS

We examined data from four EDs between March 2018–April 2023 in three adult EDs (AED) and one pediatric ED (PED) with total pre-COVID-19 annual census >250,000 patients. Each ED provided data on monthly ED census during the time period of the study, 23 months of data pre COVID-19 (March 2018–January 2020), 25 months of data during COVID-19 (February 2020–February 2022), and 14 months after COVID-19 (March 2022–April 2023). This study included four EDs with similar patient populations but different organizational structures. One ED was a PED with nearly 55,000 visits per year prior to COVID-19. The three AEDs included a suburban community ED of approximately 33,000 visits pre-COVID-19 that is a primary stroke center with an admission rate of 18%; a mixed academic/community ED of nearly 75,000 visits pre-COVID-19 that is a primary stroke center/STEMI angiography center with an admission rate of 24%; and a Level I academic urban trauma center/comprehensive stroke center with over 100,000 visits pre-COVID-19 with an admission rate of 26%.

We compared four methods of monthly volume forecasting: simple exponential smoothing with a 24-month run-up (SES); Microsoft Excel’s AAA version of the exponential smoothing (ES) algorithm seasonally adjusted with a 24-month run-up; and Holt-Winter TES using 12- and 24-month run-up, both seasonally adjusted. The SES and ES models use the Excel function FORECAST.ETS, which calls for a target date, historical values for forecasting, a timeline, and seasonality (for the ES model). Holt-Winter TES models use historical data, seasonally adjusted level, and seasonality from historical data to forecast ED volume. Holt-Winter has three smoothing constants: alpha (weighting of forecast placed on recent observations); beta (weighting of forecast placed on the trend slope of recent observations); and gamma (weighting of forecast placed on the seasonality of recent observations).

We assessed the comparison of the accuracy of all four models using the root mean squared errors (RMSE), which determines how well the forecasted values fit with the observed values, the lowest RMSE being the best fitting model. Accuracy of the model was assessed using the MAPE, mean square errors (MSE) and mean absolute deviation (MAD), comparing actual to predicted monthly volume. An acceptable level of MAPE for this study was set within one standard deviation above the average MAPE for all forecasting models from the four sites. Using this approach the acceptable level of MAPE was 5.8%. Statistical analyses were conducted using Excel (Microsoft Corporation, Redmon, WA). Accuracy of the four forecasting models pre-, during, and post-COVID-19 was assessed by counting the number of months where the forecasting was at an acceptable level of MAPSE (5.8%) divided by the total of months’ forecast. This was conducted for each AED and PED site and aggregated across all sites. This study received institutional review board approval with waiver of consent.

## RESULTS

In the 23 months prior to COVID-19, the overall average MAPE across four forecasting methods was 3.88% ± 1.88% (range of 2.41% to 6.42% across the four ED sites). The overall average MAPE for the 25 months during COVID-19 pandemic was 15.21% ± 6.67% during COVID-19 (range of 9.97–25.18% across the four ED sites). In the 14 months following COVID-19, the overall average MAPE was 6.45% ± 3.92% after (range of 3.86–12.34% across the four ED sites). Due to the large difference in forecasting MAPE and accuracy across all time points of interest for the PED, performance was focused on the three AEDs.

**Figure 1.**
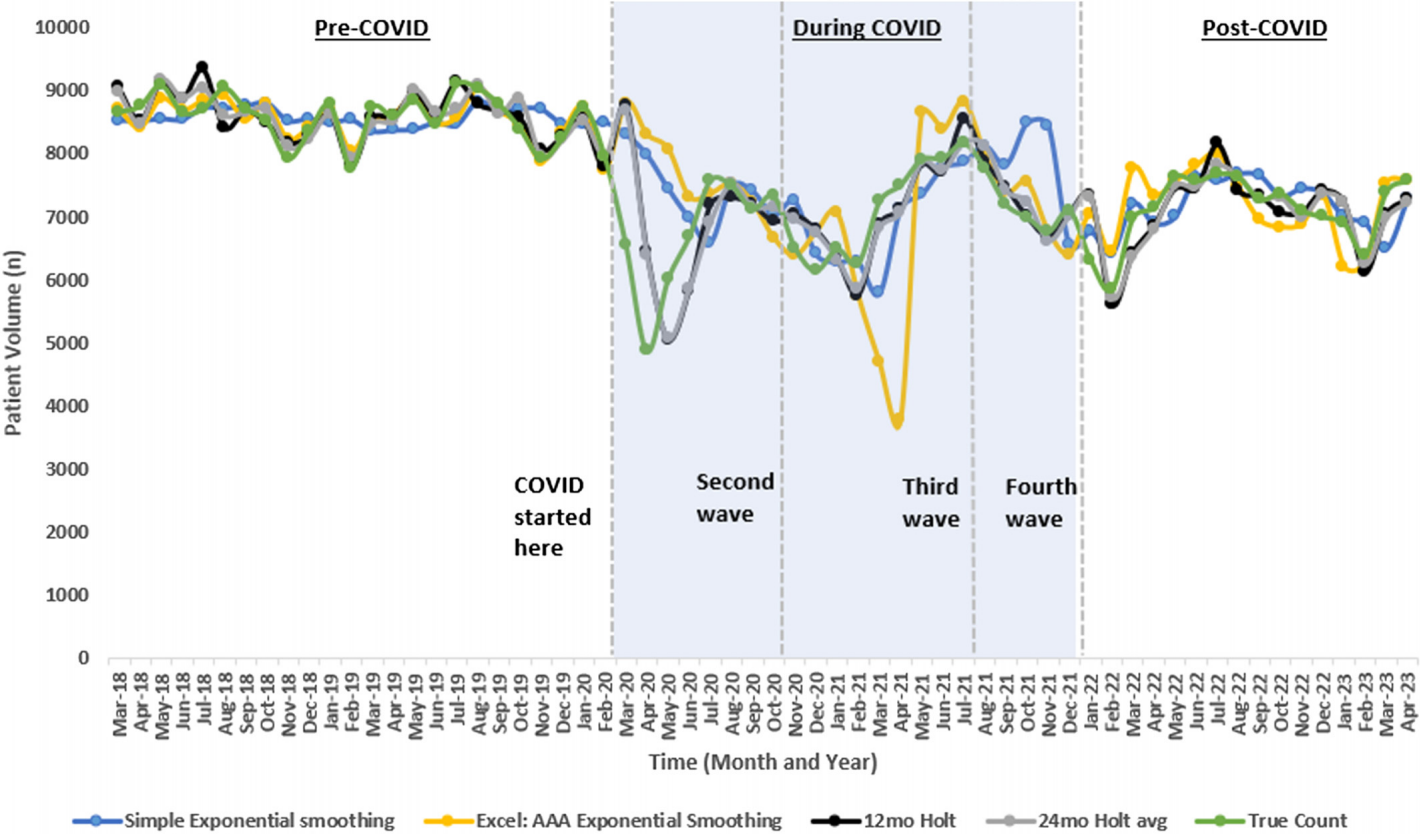
Adult emergency department #1 trend in actual and forecasted patient volume over time. *COVID-19*, coronavirus 2019.

Defining an acceptable limit to the MAPE as 1 SD above the upper range pre-COVID-19 MAPE (5.8%) resulted in the overall average of all forecasting models across the three AED sites being accurate was 49% of months *during* COVID-19 (range of 42–51% for overall forecasting model accuracy) and 71.43% *after* COVID-19 (range of 64.29–82.14% for overall forecasting model accuracy). (See Table 1). Following the COVID-19 pandemic, the overall Holt-Winter TES models indicated improvements trending toward pre-COVID-19 accuracy as observed with reductions in the MAPE and improvements in forecasting accuracy within the acceptable limits of the MAPE. Among all AEDs, the 12-month Holt-Winter had the greatest accuracy prior to COVID-19 (overall 2.36% ± 0.46%) and during the pandemic (8.92% ± 1.5%), while the 24-month Holt-Winter offered the best performance following the pandemic (3.98% ± 0.9%) (Table 1). Interestingly, for AED site 1 post COVID-19, a significantly larger accuracy was obtained using the Holt-Winter 24-month model (98.26%, 95% confidence interval (CI) 89.25–96.46%). This AED has the largest patient census. One example of this shift in the dynamic visualizing the different forecasting models for one AED site (site #1) is shown in Figure 1.

**Table 1.** Four forecasting models mean average percentage error and accuracy across three adult emergency departments.

	Pre-COVID-19 MAPE^1^% (95% CI)	During COVID-19 MAPE^2^% (95% CI)	Accuracy during COVID-19 /25 months% (95% CI)	Post-COVID-19 MAPE^3^% (95% CI)	Accuracy post- COVID-19 /14 months% (95% CI)
Site 1					
SES	3.68 (3.67–3.69)	11.52 (11.46–11.59)	44 (40.11–47.89)	4.29 (4.27–4.31)	78.57 (72.83–84.32)
Excel AAA	1.62 (1.61–1.63)	13.85 (13.77–13.93)	36 (32.24–39.76)	4.14 (4.12–4.16)	78.57 (72.83–84.32)
HW 12 m	2.04 (2.03–2.05)	7.40 (7.36–7.45)	68 (64.34–71.66)	3.85 (3.84–3.87)	78.57 (72.83–84.32)
HW 24 m	2.31 (2.3–2.31)	7.09 (7.04–7.13)	56 (52.11–59.89)	3.14 (3.13–3.16)	92.86 (89.25–96.46)
Average	2.41 (1.54–3.29)	9.97 (6.75–13.18)	51 (47.08–54.92)	3.86 (3.35–4.36)	82.14 (76.78–87.5)
Site 2					
SES	3.72 (3.71–3.74)	14.51 (14.41–14.62)	44 (40.11–47.89)	5.23 (5.2–5.26)	57.14 (50.21–64.07)
Excel AAA	2.45 (2.44–2.47)	18.65 (18.53–18.78)	28 (24.48–31.52)	4.15 (4.13–4.16)	78.57 (72.83–84.32)
HW 12 m	2.21 (2.2–2.22)	9.84 (9.77–9.91)	52 (48.08–55.92)	4.12 (4.09–4.15)	78.57 (72.83–84.32)
HW 24 m	2.36 (2.35–2.37)	10.61 (10.53–10.68)	44 (40.11–47.89)	4.09 (4.07–4.12)	71.43 (65.1–77.75)
Average	2.69 (2.0–3.37)	13.4 (9.43–17.37)	42 (38.13–45.87)	4.4 (3.85–4.94)	71.43 (65.1–77.75)
Site 3					
SES	7.64 (7.61–7.68)	12.95 (12.84–13.07)	36 (32.24–39.76)	7.20 (7.16–7.24)	42.86 (35.93–49.79)
Excel AAA	2.46 (2.44–2.48)	17.58 (17.38–17.77)	36 (32.24–39.76)	4.50 (4.46–4.54)	71.43 (65.1–77.75)
HW 12 m	2.82 (2.81–2.83)	9.51 (9.41–9.62)	56 (52.11–59.89)	4.37 (4.34–4.41)	71.43 (65.1–77.75)
HW 24 m	3.09 (3.07–3.1)	9.08 (8.97–9.19)	56 (52.11–59.89)	4.72 (4.68–4.76)	71.43 (65.1–77.75)
Average	4.0 (1.61–6.39)	12.28 (8.42–16.14)	46 (42.09–49.91)	5.2 (3.88–6.51)	64.29 (57.58–70.99)
AED Average					
SES	5.01 (2.44–7.59)	12.99 (11.3–14.69)	32 (28.34–35.66)	5.57 (3.89–7.25)	57.14 (50.21–64.07)
Excel AAA	2.18 (1.63–2.72)	16.69 (13.84–19.54)	40 (36.16–43.84)	4.26 (4.03–4.5)	71.43 (65.1–77.75)
HW 12 m	2.36 (1.89–2.82)	8.92 (7.42–10.41)	64 (60.24–67.76)	4.11 (3.81–4.41)	78.57 (72.83–84.32)
HW 24 m	2.59 (2.09–3.08)	8.93 (6.93–10.92)	60 (56.16–63.84)	3.98 (3.08–4.88)	78.57 (72.83–84.32)
Average	3.03 (2.01–4.05)	11.89 (9.87–13.89)	49 (45.08–52.92)	4.48 (3.71–5.26)	71.43 (65.1–77.75)

1 Pre-COVID-19: includes ED volume from March 2018–January 2020.

2 During-COVID-19: includes ED volume from February 2020–February 2022.

3 Post-COVID-19: includes ED volume from March 2022–April 2023.

*COVID-19*, coronavirus 2019; *CI*, confidence interval; *MAPE*, mean average percentage error; *AED*, adult emergency department; *SES*, simple exponential smoothing; *HW 12 m*, Holt-Winter 12 month; *HW 24*, Holt-Winter 24 month.

The PED was consistently less accurate than the AED, with an average MAPE of 6.42% ± 1.54% prior to COVID-19, 25.18% ± 9.42% during the pandemic, and 12.34% ± 0.55% after COVID-19. When combining all four EDs, Holt-Winter models accuracy decreased from 3.18% ± 1.65 pre-COVID-19, 11.31% ± 4.81 during the pandemic and 6.16% ± 4.02 after COVID-19 for the 12-month model and decreased from 3.37% ± 1.57 pre-COVID-19 11.51% ± 5.25 during the pandemic, and 5.91% ± 3.82 after COVID-19 for the 24-month model.

## DISCUSSION

Large-scale healthcare institutional decisions have been substantially improved by the introduction of predictive analytics in healthcare. Until recently, forecasting ED volumes has been subjective and largely determined by consensus estimates of hospital and ED leadership.^2^ ^,^ ^10^ Using consensus opinion for forecasting volumes is most applicable in settings where institutional leadership has some control over the volume estimates and the variability is lower. Predictive analytics improve accuracy when variability is too complex for subjective estimation, where volatility may be high, and when volume of services to be rendered is out of control of the institutional leadership. Therefore, predicting ED volumes should be ideally suited for these analytical methods. Recent work has demonstrated that predictive analytics can be used to forecast ED volumes with some degree of accuracy,^1^ ^–^ ^3^ ^,^ ^5^ ^–^ ^7^ ^,^ ^9^ although a 5-9% mean absolute percentage error is unacceptable for financial and logistical planning.

Our study yields several interesting findings. First, we were able to find a forecasting methodology that was superior and yielded pre-COVID-19 forecasting accuracy better than was previously reported in the literature. Our 12-month Holt-Winter TES model had a MAPE of 3.18% ± 1.65% across all four EDs. This is nearly 2–3 times better than previously reported in the literature.^1^ ^,^ ^2^ ^,^ ^4^ ^–^ ^6^ Our four EDs have a combined census of approximately 250,000 visits; prior to the pandemic this would have meant TES model accuracy within ±5,000 patients. One notable exception is that pre-, during and post-COVID-19 estimates using TES in the PED demonstrated MAPE twice that of the AED counterparts (Table 1).

Under normal circumstances, this model would be an excellent one to augment or perhaps supplant subjective consensus opinion. However, the COVID-19 pandemic disrupted normal assumptions about ED volume estimates and significantly altered the forecasting landscape using predictive analytics. After the onset of the pandemic, the accuracy of this model was significantly upended and resulted in substantial reductions in accuracy. Since onset of the pandemic, the MAPE is 4–5 times larger. A MAPE of about 10% would not be tolerated and would not be considered more accurate than subjective estimates by administrative leadership. This is particularly the case in the PED, which demonstrated a MAPE nearly twice that of the AEDs. COVID-19 hampered the ability of the TES model to accurately forecast ED volume, but post-COVID-19 has shown promise. Applying previous standards of accuracy (pre-COVID-19) demonstrates the models (post-COVID-19) were, on aggregate, able to accurately predict AED volume more than 70% of the time (71.43%). Although the three models with seasonal adjustment outperformed the SES model with no seasonal adjustment, this post-COVID-19 accuracy was an improvement from the 49% accuracy of the forecasting model during-COVID-19. On the contrary, overall PED volume accuracy remained poor during-COVID-19 (17%) and post-COVID (23.21%).

## LIMITATIONS

There are several limitations to our study. While this study included four EDs in the same region, it includes four departments of varying size and demographics. However, each of these EDs demonstrated the same pattern, with pediatrics showing even greater changes in accuracy. While further stability in the model might be expected over time, recurrent COVID-19 surges have not shown a predictable pattern (Figure 1), making it doubtful that forecasting will substantially improve in the short term. The data abstractors were not blinded to the study hypothesis; however, they were abstracting objective data and were not involved in the data analysis. Lastly, although the changes brought on by the COVID-19 pandemic were similar across all four of our EDs, regional variations are likely to exist, particularly based on the effect that COVID-19 surges have had on ED volumes.

## CONCLUSION

Under normal operating circumstances triple exponential smoothing represents an improvement in the accuracy of ED volume prediction. However, the COVID-19 pandemic significantly upset this balance, resulting in accuracy levels that are 4–5 times worse than they once were. During the pandemic, even the most accurate TES method was only able to meet pre-COVID-19 predictive accuracy levels approximately 64% of the time. Fortunately, after the pandemic, the forecasting ability did improve with predictive levels approximately 78.57% of the time. Hospital and ED operations leadership need to take this into account when forecasting budgetary needs. Future work is needed that confirms this decrease in forecasting accuracy and potentially forecast when these models will return to baseline levels of accuracy.
